# Effect of pelvic floor muscle training on reports of urinary incontinence in obese women undergoing a low-calorie diet before bariatric surgery — protocol of a randomized controlled trial

**DOI:** 10.1186/s13063-023-07347-4

**Published:** 2023-06-05

**Authors:** Pauliana C. S. Mendes, Tatiana B. Fretta, Milena F. C. Camargo, Patricia Driusso, Cristine Homsi Jorge

**Affiliations:** 1 Department of Health Sciences, University of Sao Paulo, Ribeirão Preto, São Paulo, Brazil; 2 University of São Paulo, Ribeirão Preto, São Paulo, Brazil; 3 Federal University of Sao Carlos, São Carlos, Brazil; 4 University of São Paulo, Ribeirão Preto Medical School, Graduation Program in Rehabilitation and Functional Performance, Avenida Bandeirantes 3900, Monte Alegre, SP 14.049-900 Ribeirão Preto, Brazil

**Keywords:** Urinary incontinence, Pelvic floor, Physiotherapy, Women, Obesity, Diet therapy

## Abstract

**Background:**

Obesity represents a growing threat to health with multiple negative impacts including urinary incontinence. Pelvic floor muscle training (PFMT) is the first line of treatment for urinary incontinence. Both surgical and conservative weight loss results in improvement of urinary incontinence reports in obese women and we hypothesize that a low-calorie diet in combination with PFMT would result in additional beneficial effects to urinary symptoms in women with UI compared would with weight loss alone.

**Objective:**

To assess the effect of a low-calorie diet plus PFMT protocol in obese women’s urinary incontinence reports.

**Methods:**

This is a protocol for a randomized controlled trial that will include obese women reporting UI and being able to contract their pelvic floor muscles. The participants will be randomly allocated in two groups: group 1 will participate in a 12-week protocol of low-calorie diet delivered by a multi-professional team at a tertiary hospital; group II will receive the same low-calorie diet protocol during 12 weeks and will additionally participate in 6 group sessions of supervised PFMT delivered by a physiotherapist. The primary outcome of the study is self-reported UI, and severity and impact of UI on women’s quality of life will be assessed by the ICIQ-SF score. The secondary outcomes will be adherence to the protocols assessed using a home diary, pelvic floor muscle function assessed by bidigital vaginal palpation and the modified Oxford grading scale, and women’s self-perception of their PFM contraction using a questionnaire. Satisfaction with treatments will be assessed using a visual analog scale. The statistical analysis will be performed by intention to treat and multivariate analysis of mixed effects will be used to compare outcomes. The complier average causal effects (CACE) method will be used to assess adherence. There is an urgent need for a high-quality RCT to investigate if the association of a low-calorie diet and PFMT can provide a larger effect in the improvement of urinary incontinence reports in women with obesity.

**Trial registration:**

Clinical Trials NCT04159467. Registered on 08/28/2021.

**Supplementary Information:**

The online version contains supplementary material available at 10.1186/s13063-023-07347-4.

## Introduction

Currently, the number of obese people in the world represents a growing threat to the health of populations, both in developed and developing countries [1]. According to the World Health Organization (WHO), in 2016, more than 1.9 billion adults aged 18 and over were overweight. Of these, more than 650 million adults were obese. In 2016, 39% of adults aged 18 and over (39% of men and 40% of women) were overweight. Overall, about 13% of the world’s adult population (11% of men and 15% of women) was obese in 2016 [2]. In addition to metabolic diseases, obesity can cause musculoskeletal disorders, including pelvic floor muscle disorders, the most prevalent of which is urinary incontinence (UI) [3–5].

Pelvic floor muscle training (PFMT) is considered the first-line treatment of all types of non-neurogenic urinary incontinence (UI) in women [6]. Studies have shown the positive effect of weight loss, mediated by a low-calorie diet on UI symptoms in women [7–9] but few current randomized controlled trials (RCT) have been found comparing the improvement in UI symptoms in women who have just undergone a low-calorie diet for weight loss compared to a low-calorie diet associated with PFMT.

Considering that obesity is currently among the most worrying health problems in the world, contributing to PFM dysfunctions such as UI, it is urgent to investigate in high-quality RCTs new low-risk interventions capable to optimize the results obtained only with weight loss, in the reduction of reports of UI in obese women.

## Methods

### Study design

We will conduct an assessor blind randomized, controlled, two-arm clinical trial to assess whether the combination of a low-calorie diet and PFMT will optimize the results obtained with diet therapy as a single intervention to decrease UI prevalence reports and severity in obese women (primary outcome). The secondary outcomes of this study are the impact of the protocol on PFM function, women’s self-perception of their PFM, adherence, and satisfaction with the treatment. This protocol will be reported according to the Protocol Items Standard: Recommendations for Intervention Trials checklist (SPIRIT) 2013: items recommended for a clinical trial protocol and related documents. It is a superiority trial.

### Ethic

The study was approved by the Human Research Ethics Committee of the University of São Paulo—Hospital das Clínicas of the Ribeirão Preto Medical School (HCFMRP / USP) No. 16379919.5.0000.5440 on July 9, 2019, and registered in the Clinical Trials—NCT04159467.

Any major deviation or modification of the original protocol will be reported in the Clinical Trials website and the trial records will be continuously updated as necessary.

The risks of this research basically include the possible embarrassment of the participant in answering questions related to symptoms of urinary incontinence, intestinal and vaginal pain, and embarrassment during the physical examination (vaginal palpation), in addition to feeling some discomfort during the examination and of the possible breach of confidentiality during the research.

The benefits of this research are that the participant will receive information about the anatomy of the pelvic floor, its functions, and dysfunctions, she will learn to contract this musculature and know if she is doing it correctly, contributing to prevent and/or treat its dysfunctions, in addition to also receiving a program of training for 12 weeks (if selected for group II) to strengthen the pelvic floor muscles. If you are selected for group I, you will be invited after 12 weeks to receive the same treatment as in group II.

For ethical reasons, we did not plan to prohibit any concomitant treatment. We included in the methods section our plan to monitor any concomitant treatment for pelvic floor disorders including urinary incontinence, but we expect to have homogeneous groups in relation to these variables provided by an adequate randomization and sample size.

**Fig. 1:**
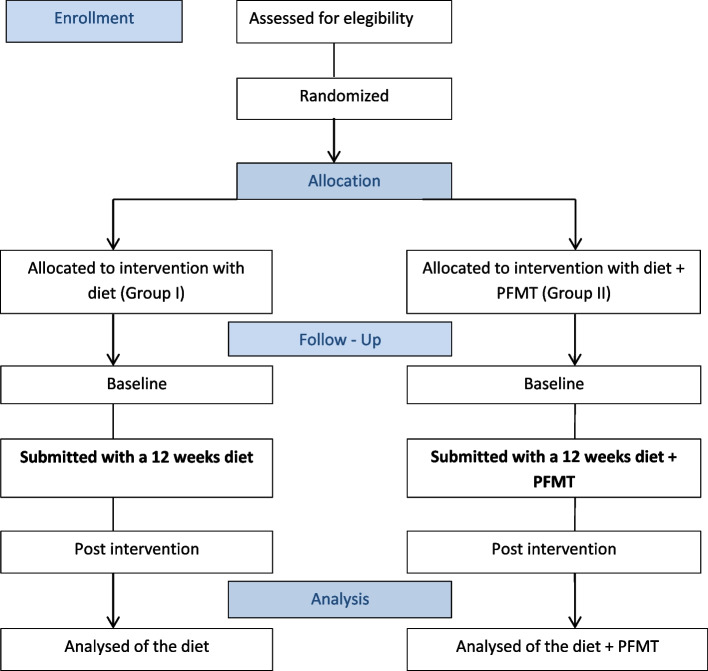
Flowchart for selecting women for the randomized clinical trial, following the CONSORT

**Fig. 2:**
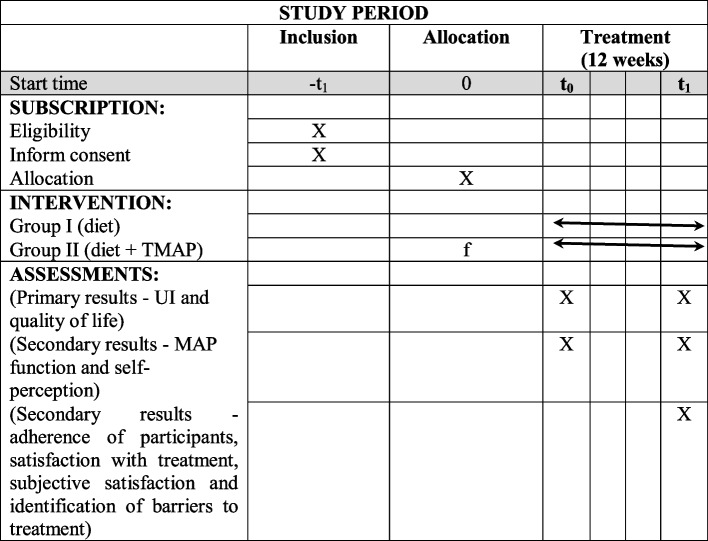
Study evaluation schedule (SPIRIT). Source: According to the SPIRIT 2013 statement: Standard Protocol Item Definition for Clinical Trials

Figure 1 shows the CONSORT (Consolidated Standards of Reporting Trials) flowchart, enrollment schedule, interventions, and study evaluations. Figure 2 shows the checklist using the SPIRIT (Standard Protocol Items: Recommendations for Interventional Trials) used in the study.

### Participants

Obese women with UI who are about to start a low-calorie diet program for weight loss will be recruited in the bariatric surgery sector at the Clinics Hospital of Ribeirão Preto Medical School (HCFMRP-USP).

### Inclusion criteria

Women over 18 years, with a body mass index (BMI) > 30 kg/m^2^; reporting stress urinary incontinence, urgent urinary incontinence, or mixed urinary incontinence in the past 4 weeks assessed by the ICIQ-SF; who are able to contract the pelvic floor muscles (assessed using vaginal palpation with a PFM contraction ≥ 2 according to the modified Oxford grading scale); and without any cognitive impairment.

### Exclusion criteria

The participants will be excluded of the study if they withdraw their consent to participate in the study or if they become pregnant.

### Criteria of discontinuing

The criteria of discontinuing the trial for a given participant include withdrawal of consent to participate and becoming pregnant during the study.

### Sample size calculation

The sample calculation was estimated considering a significance level of 5%, power of 20%, and difference of 4 points on the final score of the primary outcome, ICIQ-SF, between groups at the end of treatment [10]. The value of 4 points is equivalent to the minimum value for change in the questionnaire score. Thus, the sample will be composed of 22 participants.

### Statistical analysis

Data will be de-identified, encoded, and stored in the database using the Excel Program. The software SPSS 22.0 will be used for the analysis of data. The description of quantitative variables will be expressed in mean, standard deviation, minimum value, median and maximum value. Qualitative variables will be described in absolute and relative values (%). A 95% confidence interval and 0.05 significance will be considered for all tests. Both per-protocol and intention-to-treat analyses will be performed.

A linear mixed-effect model will be used to analyze the ICIQ-SF score. In the reassessment, individuals’ values will be considered random effects, and the groups, the times, and the interaction between them will be regarded as fixed effects. In addition, the McNemar test will verify changes in categories variables before and after the intervention. Both per-protocol analysis [11–13] and an intention-to-treat analysis will be also conducted.

### Randomization and blinding process

An assistant researcher specially trained will be responsible only for recruiting the participants to enter the study and will give them all the information about the study, obtaining their written consent in case they agree to participate.

An assistant researcher not involved with the recruitment, intervention and assessment will generate the allocation sequence. The participants will be allocated in a group that will receive a low-calorie diet for weight loss (group I) or in another group that will receive diet + PFMT (group II). Randomization will be carried out using a computational list of random numbers and will be concealed (by means of a sealed brown envelope). Women’s data will be kept only with the assistant researcher not involved with other parts of the research process to process to protect confidentiality before, during, and after the intervention. The trial is an assessor blinded in relation to the pelvic floor muscle assessment.

The senior researcher who is not involved with the recruitment, intervention delivery, and assessment will have access to the trial data. We have planned to submit the manuscript to an open-access journal that makes all data available to readers as an appendix or under request.

Figure 1 shows the flowchart for selecting women for the randomized clinical trial, following the CONSORT.

### Intervention

Women randomized to Group 1 will receive counseling from the hospital staff. They will attend 3 meetings (once a month), where a multi-professional team from the bariatric surgery outpatient clinic will address different topics related to nutrition including advice on the quality of food, mindful eating, identification of signs of hunger and satiety, and emotional triggers that lead to emotional eating. In addition, each participant will undergo individual nutritional assistance to assess food consumption, anthropometric measures, and body composition and will receive more specific guidelines on nutrition and an individualized low-calorie diet and monitoring. Outpatient care will last 12 weeks and women in this group will not receive instructions and a supervised PFMT protocol, however for ethical reasons, after the data collection is finished women belonging to this group will be invited to receive six supervised sessions of PFMT and will receive the same booklet of exercises on PFMT delivered to group II.

The participants randomized to group II will also receive the same counseling related to nutrition and also an individualized low-calorie diet given by the hospital staff; additionally, they will participate in six face-to-face physiotherapy sessions supervised by a physiotherapist in groups of a maximum of 10 participants, for 12 weeks in interspersed weeks.

In the weeks when there is no face-to-face supervised session, the same physiotherapist will encourage home PFMT by phone, calling each participant of Group II only. An intensive PFMT will be encouraged by the physiotherapist. The PFMT protocol will consist of 4 sets of ten maximum perceived voluntary contractions sustained for 6 s with a 6-s resting period between the contractions. At the end of a series of 10 contractions, five rapid contractions of the PFM will be performed. Two minutes interval will be given between each set performed.

The sets will be performed only in two positions at the face-to-face supervised sessions at the hospital: sitting and standing (2 sitting sets + 2 standing sets). The participants of this group will be instructed to perform PFMT at home at least three times a week, except on the days of supervised training. They will be instructed to follow the booklet given to them in the first meeting, in addition to the fortnightly adherence diary to the exercises to be filled weekly. Participants will also be instructed on how to perform the “the knack” maneuver, which consists of repeatedly contracting their PFM before any increase in intra-abdominal pressure during daily activities [14]. Women will also be instructed to repeatedly contract their PFM when they feel urgency. Women will fill a diary about their adherence to PFMT, and there will be a place where they are asked about any side effects or bother related to the intervention. At the last assessment, they are asked about again about any unintended effects related to the trial intervention. The type and number of unintended effects will be fully reported as an important result of the trial. Although adverse effects of these type of intervention is not very common or severe, all the participant is assured they can discontinue their participation at any time for any reason, including any adverse effects (Table 1). Table 1 presents detailed information about the protocol of intervention.

**Table 1:** Stages of the study and information about the 12 weeks training

	Recruitment	
	1^st^ Physical therapy evaluation	
	Randomization	
Week 1	Face-to-face session	4 sets (2 sitting sets + 2 standing sets) of ten maximum perceived voluntary contractions sustained for 6 s with a 6-s resting period between the contractions. At the end of a series of 10 contractions, five rapid contractions of the PFM will be performed. Two minutes interval will be given between each set performed in each position
Week 2	Face-to-face session	4 sets (2 sitting sets + 2 standing sets) of ten maximum perceived voluntary contractions sustained for 6 s with a 6-s resting period between the contractions. At the end of a series of 10 contractions, five rapid contractions of the PFM will be performed. Two minutes interval will be given between each set performed in each position
Week 3	Face-to-face session	4 sets (2 sitting sets + 2 standing sets) of ten maximum perceived voluntary contractions sustained for 6 s with a 6-s resting period between the contractions. At the end of a series of 10 contractions, five rapid contractions of the PFM will be performed. Two minutes interval will be given between each set performed in each position
Week 4	Face-to-face session	4 sets (2 sitting sets + 2 standing sets) of ten maximum perceived voluntary contractions sustained for 6 s with a 6-s resting period between the contractions. At the end of a series of 10 contractions, five rapid contractions of the PFM will be performed. Two minutes interval will be given between each set performed in each position
Week 5	Face-to-face session	4 sets (2 sitting sets + 2 standing sets) of ten maximum perceived voluntary contractions sustained for 6 s with a 6-s resting period between the contractions. At the end of a series of 10 contractions, five rapid contractions of the PFM will be performed. Two minutes interval will be given between each set performed in each position
Week 6	Face-to-face session	4 sets (2 sitting sets + 2 standing sets) of ten maximum perceived voluntary contractions sustained for 6 s with a 6-s resting period between the contractions. At the end of a series of 10 contractions, five rapid contractions of the PFM will be performed. Two minutes interval will be given between each set performed in each position
Week 7	Face-to-face session	4 sets (2 sitting sets + 2 standing sets) of ten maximum perceived voluntary contractions sustained for 6 s with a 6-s resting period between the contractions. At the end of a series of 10 contractions, five rapid contractions of the PFM will be performed. Two minutes interval will be given between each set performed in each position
Week 8	Face-to-face session	4 sets (2 sitting sets + 2 standing sets) of ten maximum perceived voluntary contractions sustained for 6 s with a 6-s resting period between the contractions. At the end of a series of 10 contractions, five rapid contractions of the PFM will be performed. Two minutes interval will be given between each set performed in each position
Week 9	Face-to-face session	4 sets (2 sitting sets + 2 standing sets) of ten maximum perceived voluntary contractions sustained for 6 s with a 6-s resting period between the contractions. At the end of a series of 10 contractions, five rapid contractions of the PFM will be performed. Two minutes interval will be given between each set performed in each position
Week 10	Face-to-face session	4 sets (2 sitting sets + 2 standing sets) of ten maximum perceived voluntary contractions sustained for 6 s with a 6-s resting period between the contractions. At the end of a series of 10 contractions, five rapid contractions of the PFM will be performed. Two minutes interval will be given between each set performed in each position
Week 11	Face-to-face session	4 sets (2 sitting sets + 2 standing sets) of ten maximum perceived voluntary contractions sustained for 6 s with a 6-s resting period between the contractions. At the end of a series of 10 contractions, five rapid contractions of the PFM will be performed. Two minutes interval will be given between each set performed in each position
Week 12	Face-to-face session	4 sets (2 sitting sets + 2 standing sets) of ten maximum perceived voluntary contractions sustained for 6 s with a 6-s resting period between the contractions. At the end of a series of 10 contractions, five rapid contractions of the PFM will be performed. Two minutes interval will be given between each set performed in each position
	2^st^ Physical therapy evaluation (Group I and II)	
	Diary about the adherence to PFMT	Women will fill a diary about their adherence to PFMT, and there will be a place where they are asked about any side effects or bother related to the intervention. At the last assessment, they are asked about again about any unintended effects related to the trial intervention. The type and number of unintended effects will be fully reported as an important result of the trial. Although adverse effects of these type of intervention are not very common or severe, all the participant is assured they can discontinue their participation at any time for any reason, including any adverse effects

### Assessment

#### Primary outcome measures

Self-report of urinary incontinence will be measured by question 3 of the ICIQ-SF. Women will be considered incontinent if they choose options 1, 2, 3, 4, or 5 of question 3. Women will be considered continent if they choose option 0 in question 3.

The severity and impact of urinary incontinence on women’s quality of life will be measured by the ICIQ-SF score.

#### Other outcome measures

The adherence to home PFMT sessions will be monitored by the physiotherapist from a diary where women will register their adherence to home training. This diary will be attached to the leaflet that will be given to women in the first session. The diary will be collected by the physiotherapist every fortnight.

The PFM function will be assessed using vaginal palpation by a trained physiotherapist who will not be involved with the intervention and will not be aware of women’s allocation in the groups. Before the start of the palpation exam, the woman will receive detailed information about the anatomy, function, and dysfunction of the PFM and how to contract them correctly. The evaluation will be carried out with the participant in the supine position with the hip and knee semi-flexed. The participant will be asked to pull their PFM in and up as hard as possible, and then she will be instructed to relax them completely. Contraction of PFM will be classified as absent or present. The ability to contract the PFM will be considered present when there is occlusion and/or occlusion and elevation of the PFM towards the pubic symphysis and absent when there is no perceived internal movement. Additionally, muscle contraction will be graded according to the Modified Oxford Grading Scale (MOGS) [15, 16]. Contractions of muscles such as gluteus, adductor, and abdominal will be discouraged by the examiner.

Self-perception of women’s PFM contraction will be assessed at the time of the physical evaluation when the examiner will ask the participant if she feels she is able to contract her PFM and she will be asked to estimate the intensity of her PFM according to the MOGS that will be presented to her [17].

Participants’ subjective satisfaction with the treatment will be measured by the visual analog scale (VAS), which is an estimate on a numerical scale where 0 represents no satisfaction at all and 10 means the maximum possible satisfaction with the treatment.

In relation to satisfaction with the interventions, participants will be asked to answer the following questions: “Are you satisfied with the treatment you received for UI?”, “Would you do this treatment again?”, “Would you recommend this treatment?”, “Would you pursue another treatment?”. The options of answers to these questions are yes or no.

In order to identify barriers to treatment, the participants will be asked to answer the following questions: “Do you identify any barriers or difficulties in adhering to the treatment you received for urinary incontinence? If any please list the barriers.”

### Descriptive and control variables

The descriptive and control variables were divided into clinical variables (diabetes, hypertension, constipation, number of pregnancies, deliveries, cesarean sections, vaginal delivery; use of contraceptives and previous pelvic surgeries) sociodemographic variables (age, education, marital status, economic, smoking occupation, use of alcoholic beverages, physical exercise and use of caffeine) and anthropometric measures (height and body mass). The descriptive and control variables will be acquired from women’s self-report. The study variables are shown in Fig. 2 and Table 2.

**Table 2:** Outcome measures and time points of the study assessment

Results	Instruments	Baseline	Post-intervention
Primary			
Urinary incontinence	“*International Consultation on Incontinence Questionnaire—Short Form*” score (ICIQ-SF)	√	√
Secondary			
Adherence of the participants	PFMT diary		√
PFM function	Bidigital vaginal palpation (MOS and Oxford modified)	√	√
Self perception	Safe Stimated based on the Modified Oxford Grading Scale	√	√
Satisfaction with treatment	Visual analog scale (VAS)		√
Subjective satisfaction	Participants will answer the following questions: are you satisfied with the treatment you received for UI? Would you do this treatment again? Would you recommend this treatment for other people? Would you pursue another treatment?		√
Identification of barriers to treatment	Participants will answer the following questions: Do you identify any barriers or difficulties in adhering to the treatment you received for urinary incontinence?		√
Diary about the adherence to PFMT			√

### Data collection

The data will be collected in an interview format using a self-reported validated questionnaire and PFM exam. A trained assistant researcher will conduct an interview and a PFM assessment. All assessments will be performed (at baseline) and after 12 weeks for both groups and will be performed by the same researcher that will be blind about women’s group allocation.

## Discussion

We presented a protocol of a RCT that will investigate the efficacy of a 12-week pelvic floor muscle training (PFMT) program added to a low-calorie diet on obese women with urinary incontinence (UI) reports. In the literature, the benefits of weight loss to improve UI is established by the literature [6] but it is yet to be evidenced if PFMT could increase the effectiveness of weight loss on UI reports in obese women.

Weight loss, both mediated by surgical and conservative interventions, is effective to improve all types of non-neurological UI (stress, urgency, or mixed) [18, 19]. In fact, a loss of 5 to 10% of weight provides a significant improvement in UI complaints, and weight loss is considered a first-line therapy.

High obesity rates are a current global reality and reports of UI by this population have a high prevalence. When compared to a normal body mass index, overweight was associated with a one-third increase in the risk of UI (relative risk = 1.35, 95% confidence interval = 1.20–1.53), while the risk was doubled in obese women (relative risk = 1.95, 95% confidence interval = 1.58–2.42).

On the other hand, weight loss will not provide an active improvement of PFM function as PFMT, the gold standard physiotherapy intervention to treat non-neurogenic UI in women. The updated Cochrane review on this topic included randomized and quasi-randomized controlled trials investigating the effect of PFMT on UI in women. The search produced 1299 records, from which 94 potentially relevant full-text articles were retrieved. The inclusion of new studies in this updated review reinforced the results showing the effectiveness of PFMT in the treatment of any type of non-neurogenic UI in women [20].

In planning our RCT protocol, we considered what is already known from the literature to optimize the PFMT effectiveness having in mind that is essential to provide a high-quality intervention based on exercise physiology. We will include only women who are able to contract their PFM, providing education to them related to PFM function, dysfunction, and how to contract correctly their PFM as part of our intensive and supervised PFMT protocol.

RCTs investigating a low calory diet associated to PFMT including only obese women are rare in the literature. One small RCT (*n*= 22) included both overweight and obese women and was different from our RCT compared PFMT + weight loss with a group receiving only PFMT. The study did not show the benefits of adding weight loss to PFMT. However, the limited sample size and the short duration of the protocol (8 weeks) might have importantly contributed to the lack of effect [10].

A recently published study aimed to implement and evaluate the feasibility, acceptability, and effectiveness of a 12-week group exercise and healthy eating program (ATHENA) both for overweight/obese women with UI [21]. The results indicated that ATHENA was feasible to implement, with a high percentage of women (97%) reporting improved UI symptoms and significant improvements in pelvic floor dysfunction and quality of life. No significant weight change was observed although significant improvements were found in body-food choice congruence. Different from the protocol planned for our study, the program investigated included only education about health eating that might have not is enough to provide weight loss. Nevertheless, these results are limited to provide a reliable answer related to the effectiveness of the intervention, as this is not a RCT.

It seems urgent to conduct high-quality RCTs to investigate the efficacy of associating PFMT with weight loss mediated by diet therapy. The protocol of this RCT was carefully planned following the quality criteria to enhance optimal internal e external validity. Additionally, we will investigate the impact of the intervention on women’s adherence to treatment, on their self-perception of their PFM, and satisfaction with the treatment. Brazilian women’s knowledge about PFM function and dysfunction is low [22, 23]. PFMT protocol must include an education component about PFM function not only to increase women’s knowledge about it but also to improve their adherence to treatment [24, 25]. We will also have the chance to identify barriers and facilitators related adherence to the proposed interventions. A recent study has shown that women with worse PFM function tend to overestimate their PFM function [13]. The self-perception of the PFM function is important for women to localize and to exercise their PFM. Another innovation of this RCT will be to analyze the impact of the intervention with and without PFMT in women’s self-perception of their PFMT and our hypothesis is that more women in the group receiving PFMT in addition to low-calorie diet will improve their PFM perception compared to the group receiving only low-calorie diet.

## Trials status

Recruitment has not yet started because the patients are in the risk group for COVID-19 and, therefore, the clinic has temporarily suspended the appointments.

## Supplementary Information

**Additional file 1.** SPIRIT 2013 Checklist: recommended items to address in a clinical trial protocol and related documents*. 
